# Distinct Determinants in HIV-1 Vif and Human APOBEC3 Proteins Are Required for the Suppression of Diverse Host Anti-Viral Proteins

**DOI:** 10.1371/journal.pone.0003963

**Published:** 2008-12-17

**Authors:** Wenyan Zhang, Gongying Chen, Anna Maria Niewiadomska, Rongzhen Xu, Xiao-Fang Yu

**Affiliations:** 1 Department of Molecular Microbiology and Immunology, Johns Hopkins Bloomberg School of Public Health, Baltimore, Maryland, United States of America; 2 Second Affiliated Hospital, School of Medicine, Zhejiang University, Zhejiang, China; 3 College of Life Science, Jilin University, Jilin, China; 4 The Sixth Hospital of Hangzhou, Zhejiang, China; AIDS Research Center, Chinese Academy of Medical Sciences and Peking Union Medical College, China

## Abstract

**Background:**

APOBEC3G (A3G) and related cytidine deaminases of the APOBEC3 family of proteins are potent inhibitors of many retroviruses, including HIV-1. Formation of infectious HIV-1 requires the suppression of multiple cytidine deaminases by Vif. HIV-1 Vif suppresses various APOBEC3 proteins through the common mechanism of recruiting the Cullin5-ElonginB-ElonginC E3 ubiquitin ligase to induce target protein polyubiquitination and proteasome-mediated degradation. The domains in Vif and various APOBEC3 proteins required for APOBEC3 recognition and degradation have not been fully characterized.

**Methods and Findings:**

In the present study, we have demonstrated that the regions of APOBEC3F (A3F) that are required for its HIV-1-mediated binding and degradation are distinct from those reported for A3G. We found that the C-terminal cytidine deaminase domain (C-CDD) of A3F alone is sufficient for its interaction with HIV-1 Vif and its Vif-mediated degradation. We also observed that the domains of HIV-1 Vif that are uniquely required for its functional interaction with full-length A3F are also required for the degradation of the C-CDD of A3F; in contrast, those Vif domains that are uniquely required for functional interaction with A3G are not required for the degradation of the C-CDD of A3F. Interestingly, the HIV-1 Vif domains required for the degradation of A3F are also required for the degradation of A3C and A3DE. On the other hand, the Vif domains uniquely required for the degradation of A3G are dispensable for the degradation of cytidine deaminases A3C and A3DE.

**Conclusions:**

Our data suggest that distinct regions of A3F and A3G are targeted by HIV-1 Vif molecules. However, HIV-1 Vif suppresses A3F, A3C, and A3DE through similar recognition determinants, which are conserved among Vif molecules from diverse HIV-1 strains. Mapping these determinants may be useful for the design of novel anti-HIV inhibitors.

## Introduction

Human cytidine deaminase apolipoprotein B mRNA-editing catalytic polypeptide-like 3G (APOBEC3G, A3G) and other APOBEC3 proteins [1] are related to a family of cytidine deaminases that also includes apolipoprotein B-editing catalytic subunit 1 (APOBEC1), APOBEC2, and activation-induced cytidine deaminase (AID) [2]–[13]. These proteins, which are unique to mammals, have cytidine deaminase activities that modify RNA or DNA. Human APOBEC3 proteins exhibit varying degrees of inhibitory activity against retroviruses, such as HIV and SIV [14]–[22]; endogenous retroviruses [23]; non-LTR retrotransposons, such as LINE1 [24]–[31] and Alu [24], [25], [31], [32]; HBV [33]–[38]; and AAV [26].

In the absence of the Vif protein, APOBEC3 proteins are packaged into HIV-1 particles through an interaction with Gag protein molecules [39]–[45] and the help of cellular 7SL RNA [46] and/or viral genomic RNA [47], [48]. Virion-packaged A3G mediates cytidine deamination in the viral minus-strand DNA during new target cell infection [19], [21], [22], [49]–[52]. Virion-packaged A3G and A3F can also reduce the accumulation of viral DNA by inhibiting reverse transcription processes [53]–[59] or inducing viral DNA degradation [60], [61]. In addition, a potent inhibitory effect of A3G on the formation of proviral DNA has been described [21], [22], [55], [56]. Whether A3G inhibits HIV-1 mainly through cytidine deamination of viral DNA is still controversial.

HIV-1 Vif suppresses the activity of multiple human APOBEC3 proteins by assembling a viral-specific E3 ubiquitin ligase through its interaction with cellular Cullin5 (Cul5)-ElonginB-ElonginC proteins [62]–[65]. Vif induces polyubiquitination of APOBEC3 proteins and tags them for proteasome-mediated degradation [62], [63], [66]–[72]. The carboxyl-terminal BC-box (the SLQxLA motif) of HIV-1 Vif recruits ElonginC and ElonginB [62]–[64], [71], and a highly conserved zinc-binding Hx_5_Cx_17–18_Cx_3–5_H motif [73]–[76] and downstream LPx_4_L motif in Vif mediate Cul5 association [77].

Various amino-terminal domains of HIV-1 Vif are responsible for its specificity in recognizing the various APOBEC3 proteins [13], [67], [78]–[83]. For example, the HIV-1 Vif region spanning amino acids 22 to 44 is important for the suppression of A3G but not A3F [78], [81], [82]. In contrast, amino acids 11 to 17 and 74 to 79 are important for the suppression of A3F but not A3G [78]–[81], [83]. A stretch of hydrophobic amino acids 55 to 72 of HIV-1 Vif (VxIPLx_4–5_LxΦx_2_YWxL) is critical for both A3G and A3F binding and suppression [78]–[81], [83]. In addition to A3G and A3F, other human cytidine deaminases such as A3C and A3DE are also subject to HIV-1 Vif-induced polyubiquitination and degradation involving Cul5-ElonginB-ElonginC [72], [84]. However, little is known about how these proteins are recognized by HIV-1 Vif.

In this study, we demonstrate that A3C and A3DE are recognized by HIV-1 Vif in a fashion similar to that observed for A3F, distinct from that seen for A3G. The carboxyl-terminal cytidine deamination domain of A3F alone is sufficient for its interaction with Vif and Vif-mediated degradation of A3F, and the requirements for the degradation of full-length A3F are the same as for its carboxyl-terminal cytidine deamination domain. Thus, the single cytidine deamination domain of A3C and carboxyl-terminal cytidine deamination domain of A3F are sufficient for Vif binding and targeted degradation, in sharp contrast to the requirement for both the amino- and the carboxyl-terminal cytidine deamination domains in the case of A3G.

## Results

### Distinct HIV-1 Vif regions are involved in the suppression of single-domain cytidine deaminase A3C and double-domain A3G

**Figure 1:**
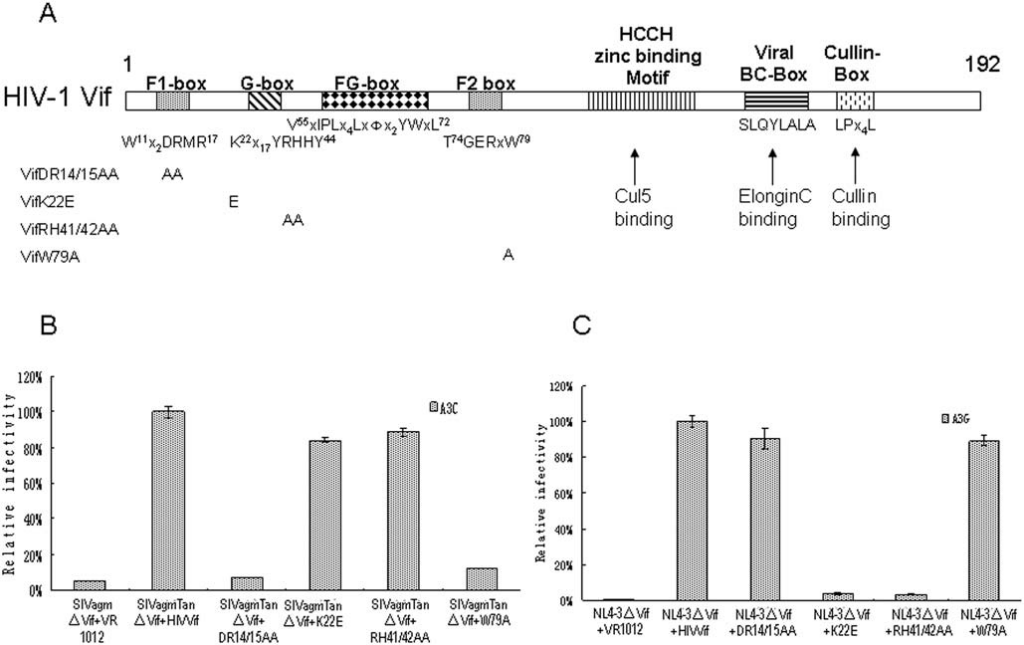
Effect of mutations in HIV-1 Vif on Vif function. (A) Diagram of the functional domains of HIV-1 Vif. The BC-box structure mediates the interaction with ElonginB/C. A zinc-binding domain, Hx2YFxCFx4Φx2AΦx7-8Cx5H, is important for Cul5 selection. The N-terminal of Vif has been proposed to bind to APOBEC3 cytidine deaminases. (B) Effect of HIV-1 WT or mutant Vif proteins on the infectivity of SIVagmTan△Vif in the presence of A3C. SIV viruses were produced in HEK293T cells co-expressing A3C in the presence of HIV-1 WT or mutant Vif as indicated. Virus infectivity was assessed by Magi assay, with virus infectivity in the presence of WT Vif set to 100%. Error bars represent the standard deviations from triplicate wells. (C) Effect of HIV-1 WT or mutant Vif proteins on the infectivity of NL4-3△Vif in the presence of A3G. HIV viruses were produced in HEK293T cells co-expressing A3G in the presence of HIV-1 WT or mutant Vif as indicated. Virus infectivity was assessed as described in Fig. 1B.

Distinct regions of HIV-1 Vif have been found to mediate A3G or A3F suppression (Fig. 1A); however, the regions of HIV-1 Vif that are involved in the suppression of other human cytidine deaminases such as A3C have not been determined. Although A3C has been shown to have only weak anti-HIV-1 activity in vitro [16], [18], it is efficiently degraded by HIV-1 Vif through the usage of Cul5-ElonginB-ElonginC E3 ubiquitin ligase. It is possible that A3C has anti-HIV-1 function in vivo that has to be neutralized by Vif to allow viral replication. Alternatively, A3C is recognized by HIV-1 Vif through a similar mechanism as other potent anti-HIV-1 cytidine deaminases such as A3G or A3F. To determine whether the previously identified regions of the Vif protein that are required for A3G or A3F inhibition are also important for its activity against A3C, we generated a series of HIV-1 Vif mutant constructs in which critical residues known to be important for A3G or A3F suppression were mutated (Fig. 1A).

It is well established that A3C is a potent inhibitor [18] of Vif-deificient simian immunodeficiency virus from African green monkeys (SIVagm) and is degraded by both SIVagm and HIV-1 Vif [85]. We therefore examined the ability of HIV-1 Vif and the various Vif mutants to suppress the anti-viral activity of A3C against SIVagm△Vif. HEK293T cells were transfected with SIVagm△Vif and with an A3C expression vector plus a control vector, an expression vector for wild-type (WT) Vif, or a Vif mutant, as indicated in Fig. 1B. Viruses were produced from the transfected cells, and viral infectivity was tested in a standard Magi assay as previously described [85], [86]. WT Vif suppressed A3C and maintained the infectivity of SIVagm△Vif (Fig. 1B, column 2); this level of viral infectivity in the presence of WT Vif was considered to be 100% for comparison purposes. As expected, A3C dramatically reduced the infectivity of SIVagm△Vif in the absence of Vif (Fig. 1B, column 1). The Vif DR14/15AA and VifW79A mutants, which have already been reported to be ineffective against A3F [79], [81], were unable to efficiently suppress the anti-viral activity of A3C (Fig. 1B, columns 3 and 6). In contrast, the Vif K22E and VifRH41/42AA mutants were able to suppress the anti-viral activity of A3C (Fig. 1B, columns 4 and 5); as expected, these two mutant proteins were ineffective in suppressing the anti-viral activity of A3G (Fig. 1C, columns 4 and 5).

### Distinct HIV-1 Vif domains are required for A3C degradation and virion exclusion

**Figure 2:**
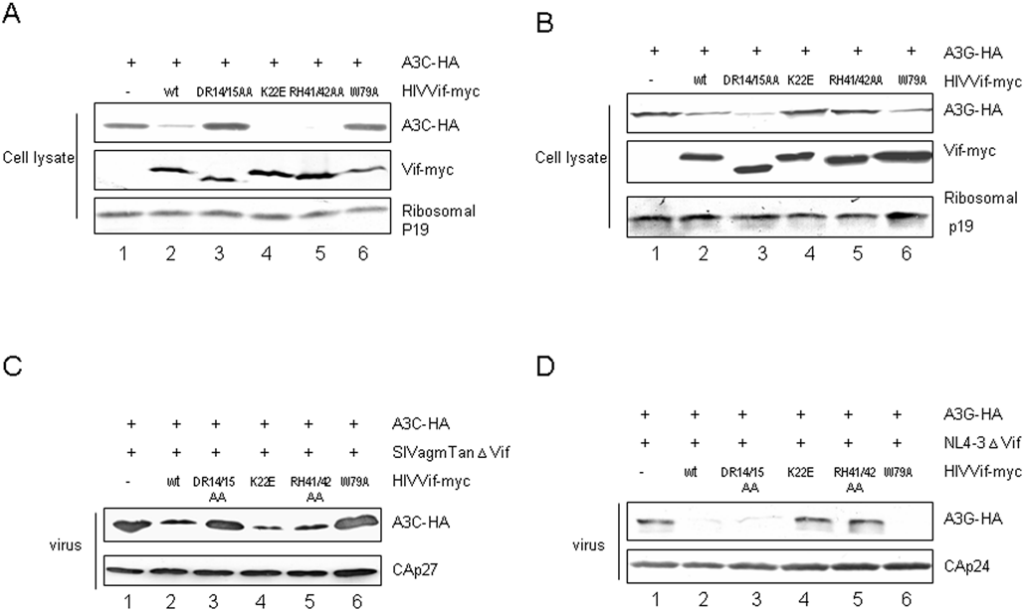
Effect of mutations in HIV-1 Vif on Vif activity against A3C and A3G. (A) Vif DR14/15 and W79 are required for A3C degradation. HEK293T cells were cotransfected with A3C plus a control vector, WT Vif, or one of the indicated Vif mutant expression vectors. A3C stability was assessed by immunoblotting against A3C-HA, Vif-myc, and ribosomal p19 as a loading control. (B) Vif K22 and RH41/42 are required for A3G degradation. HEK293T cells were cotransfected with A3G plus a control vector, WT Vif, or one of the indicated Vif mutant expression vectors. A3G stability was assessed as described in Fig. 2A. (C) Mutation of Vif DR14/15 and W79 inhibits Vif function, resulting in the packaging of A3C into SIV virions. HEK293T cells were co-transfected with SIVagmTanΔVif, A3C plus a control vector, WT Vif, or one of the indicated Vif mutants. Virus was purified from the supernatant and evaluated for A3C packaging by immunoblotting with antibodies against A3C-HA and CAp27. (D) Mutation of Vif K22 and RH41/42 inhibits Vif function, resulting in the packaging of A3G into HIV-1 virions. HEK293T cells were co-transfected with NL4-3ΔVif and A3G plus a control vector, WT Vif, or one of the indicated Vif mutants. Virus was purified and evaluated by immunoblotting with antibodies against A3G-HA and CAp24.

We also examined the effect of WT and mutant Vif molecules on the expression and virion exclusion of A3C. For this purpose, we transfected HEK293T cells with an A3C expression vector plus a control vector (Fig. 2A, lane 1), WT Vif-myc expression vector (lane 2), or one of the mutant vectors: VifDR14/15AA (lane 3), VifK22E (lane 4), VifRH41/42AA (lane 5), or VifW79A (lane 6). Consistent with the viral infectivity data (Fig. 1B), the intracellular level of A3C was efficiently reduced in the presence of WT HIV-1 Vif (Fig. 2A, lane 2) when compared to the control vector (Fig. 2A, lane 1). Mutant VifDR14/15AA (Fig. 2A, lane 3) and VifW79A (Fig. 2A, lane 6) were less effective in reducing the stability of A3C than was WT Vif (Fig. 2A, lane 2). Consequently, these two mutants were also less effective in excluding A3C from virions (Fig. 2C, lanes 3 and 6) than was the WT Vif (Fig. 2C, lane 2). Mutant VifK22E and VifRH41/42AA did not produce any significant decrease in the ability of Vif to alter A3C expression (Fig. 2A, lanes 4 and 5) or virion exclusion (Fig. 2C, lanes 4 and 5).

Collectively, these results indicate that W79 and D14R15 of HIV-1 Vif are important for Vif-mediated degradation of A3C, its exclusion from virions, and the suppression of its anti-viral activity. However, these residues were not important for Vif-mediated degradation of A3G (Fig. 2B) or its exclusion from virions (Fig. 2D). In contrast, K22 and R41H42 of HIV-1 Vif were important for Vif-mediated degradation of A3G (Fig. 2B) and its exclusion from virions, (Fig. 2D) but were dispensable for Vif-mediated A3C degradation (Fig. 2A) and virion exclusion (Fig. 2C).

### Residues in HIV-1 Vif that are required for A3F binding are also required for A3C binding

**Figure 3:**
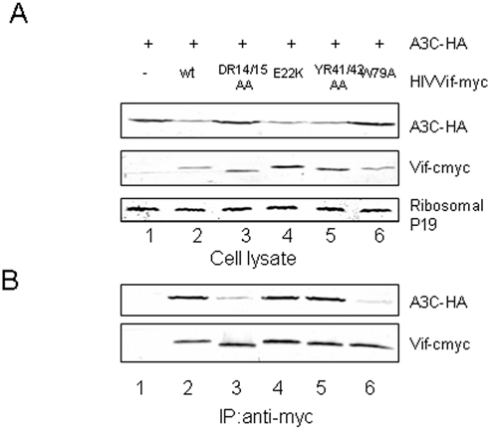
The D^14^RMR^17^ and Trp79 domains mediate the interaction between HIV-1 Vif and A3C. Vif DR14/15 and W79 showed reduced interaction with A3C when compared to WT Vif. HEK293T cells were cotransfected with A3C and the control vector, HIV-1 Vif, or one of the indicated Vif mutants. At 48 h post-transfection, cell lysates were prepared and immunoprecipitated with anti-myc antibody and agarose-conjugated protein A/G. Cell lysate (A) and the interaction of A3C with WT or mutant Vif molecules(B) were detected by immunoblotting with antibodies against A3G-HA and Vif-myc.

We then evaluated the interaction of WT and mutant Vif molecules with A3C by co-immunoprecipitation analysis. HEK293T cells were transfected with an A3C-HA expression vector plus a control vector (Fig. 3A, lane 1) or an expression vector for WT Vif-myc (lane 2), VifDR14/15AA-myc (lane 3), VifK22E-myc (lane 4), VifRH41/42AA-myc (lane 5), or VifW79A-myc (lane 6). Vif-myc proteins were immunoprecipitated from the cell lysates, and co-precipitation of A3C-HA was detected by immunoblotting. WT Vif-myc efficiently co-immunoprecipitated A3C-HA (Fig. 3B, lane 2); this interaction was specific, since A3C-HA was not detected in the absence of Vif (Fig. 3B, lane 1). Less A3C-HA was co-precipitated with VifDR14/15AA-myc (Fig. 3B, lane 3) and VifW79A-myc (Fig. 3B, lane 6) than with WT Vif-myc (Fig. 3B, lane 2), despite the fact that A3C-HA levels were higher in cells expressing VifDR14/15AA-myc (Fig. 3A, lane 3) and VifW79A-myc (Fig. 3A, lane 6) than in those expressing WT Vif-myc (Fig. 3A, lane 2). These data suggest that the impaired ability of the VifDR14/15AA and VifW79A mutants to degrade and suppress A3C is due at least in part to their reduced interaction with A3C. VifRH41/42AA-myc and VifK22E-myc were able to efficiently interact with A3C-HA (Fig. 3B, lanes 4 and 5), as compared to the WT Vif-myc (Fig. 3B, lane 2).

### Identification of the HIV-1 Vif regions required for A3DE suppression

**Figure 4:**
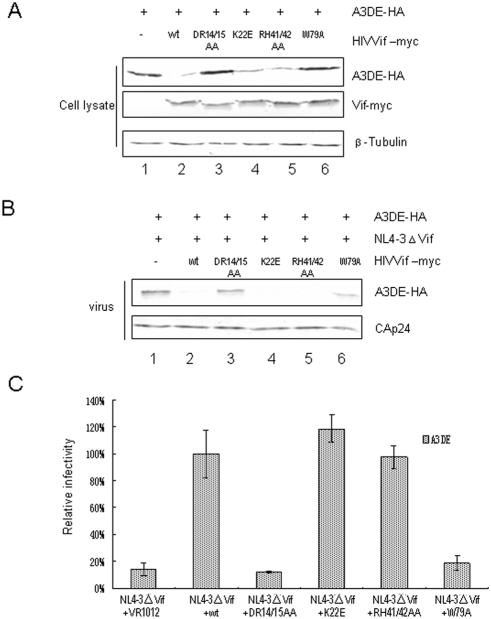
Effect of mutation of HIV-1 Vif on its activity against A3DE. (A) Vif DR14/15 and W79 are required for A3DE degradation. HEK293T cells were cotransfected with A3DE plus a control vector, HIV-1 Vif, or one of the indicated Vif mutant expression vectors. A3DE stability was assessed as described in Fig. 2A. (B) Mutation of Vif DR14/15 and W79 inhibits Vif function, resulting in the packaging of A3DE into HIV-1 virions. HEK293T cells were co-transfected with NL4-3ΔVif and A3DE plus a control vector, WT Vif, or one of the indicated mutant expression vectors. Virus was purified and evaluated for A3DE packaging as described in Fig. 2D. (C) Effect of WT or mutant Vif on the infectivity of NL4-3△Vif in the presence of A3DE. HIV viruses were produced in HEK293T cells coexpressing A3DE in the presence of WT or mutant Vif as indicated. Virus infectivity was assessed as described in Fig. 1B.

A3DE also has anti-HIV-1 activity and is neutralized by HIV-1 Vif. We next examined the ability of WT Vif and Vif mutant molecules to affect A3DE expression (Fig. 4A).To this end, we transfected HEK293T with the A3DE expression vector plus a control vector (Fig. 4A, lane 1), the expression vector for WT Vif-myc (lane 2), or one of the Vif mutant molecules (lanes 3, 4, 5, and 6). The intracellular level of A3DE was efficiently reduced by WT Vif (Fig. 4A, lane 2) when compared to the vector control (Fig. 4A, lane 1). As compared to WT Vif, VifDR14/15AA and VifW79A were less effective in reducing A3DE expression (Fig. 4A, lanes 3 and 6). However, VifK22E and VifRH41/42AA preserved the ability to reduce A3DE expression (Fig. 4A, lanes 4 and 5).

As expected, VifDR14/15AA and VifW79A (Fig. 4B, lanes 3 and 6) were less effective than WT (Fig. 4B, lane 2) in excluding A3DE from HIV-1 virions. Mutant VifDR14/15AA and VifW79A also showed a reduced ability to neutralize the anti-viral activity of A3DE (Fig. 4C, columns 3 and 6) when compared to the WT Vif (Fig. 4C, column 2). Mutant VifK22E and VifRH41/42AA were as effective as WT Vif (Fig. 4B) in excluding A3DE from virions, and they could efficiently counteract the anti-viral activity of A3DE (Fig. 4C). These data indicate that the residues of HIV-1 Vif that are important for A3F and A3C suppression are also important for A3DE suppression. Conversely, the residues of HIV-1 Vif that are important for A3G suppression are dispensable for A3DE suppression.

**Figure 5:**
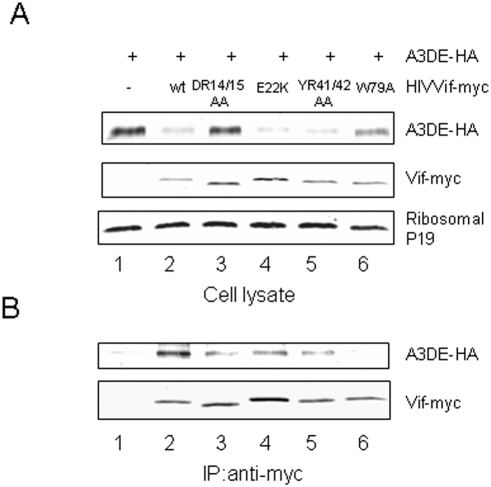
The D^14^RMR^17^ and Trp79 domains mediate the interaction between Vif and A3DE. HIV-1 Vif DR14/15 and W79 all showed reduced interaction with A3DE when compared to WT Vif. HEK293T cells were cotransfected with A3DE and a control vector, WT Vif, or one of the indicated Vif mutants. At 48 h post-transfection, cell lysates were prepared and immunoprecipitated with anti-myc antibody and agarose-conjugated protein A/G. Cell lysates (A) and the interaction of A3C with WT or mutant Vif molecules(B) were detected by immunoblotting with antibodies against A3DE-HA and Vif-myc.

We also evaluated the interaction of WT and Vif mutant molecules with A3DE by co-immunoprecipitation analysis. HEK293T cells were transfected with an A3DE expression vector plus a control vector (Fig. 5A, lane 1) or the expression vector for WT Vif-myc (lane 2), VifDR14/15AA-myc (lane 3), VifK22E-myc (lane 4), VifRH41/42AA-myc (lane 5), or VifW79A-myc (lane 6). The myc-tagged Vif proteins were immunoprecipitated from cell lysates and the co-precipitation of A3DE was assessed by immunoblotting. A3DE-HA was efficiently co-immunoprecipitated with WT Vif-myc (Fig. 5B, lane 2), and this interaction was specific, since A3DE-HA was not precipitated in the absence of Vif (Fig. 5B, lane 1). Even though higher levels of A3DE were detected in cells expressing VifDR14/15AA (Fig. 5B, lane 3) and VifW79A (Fig. 5B, lane 6) than in those expressing WT Vif, significantly less A3DE was immunoprecipitated with VifDR14/15AA and VifW79A than with WT Vif. When intracellular level of A3DE was considered, VifDR14/15AA and VifW79A had 5–10 fold reduced ability to interact with A3DE compared to the WT Vif. Thus, the impaired ability of VifDR14/15AA and VifW79A to degrade A3DE could be attributed, at least in part, to their reduced recognition of A3DE.

### The carboxyl-terminal deamination domain of A3F behaves like the full-length A3F in terms of Vif sensitivity

**Figure 6:**
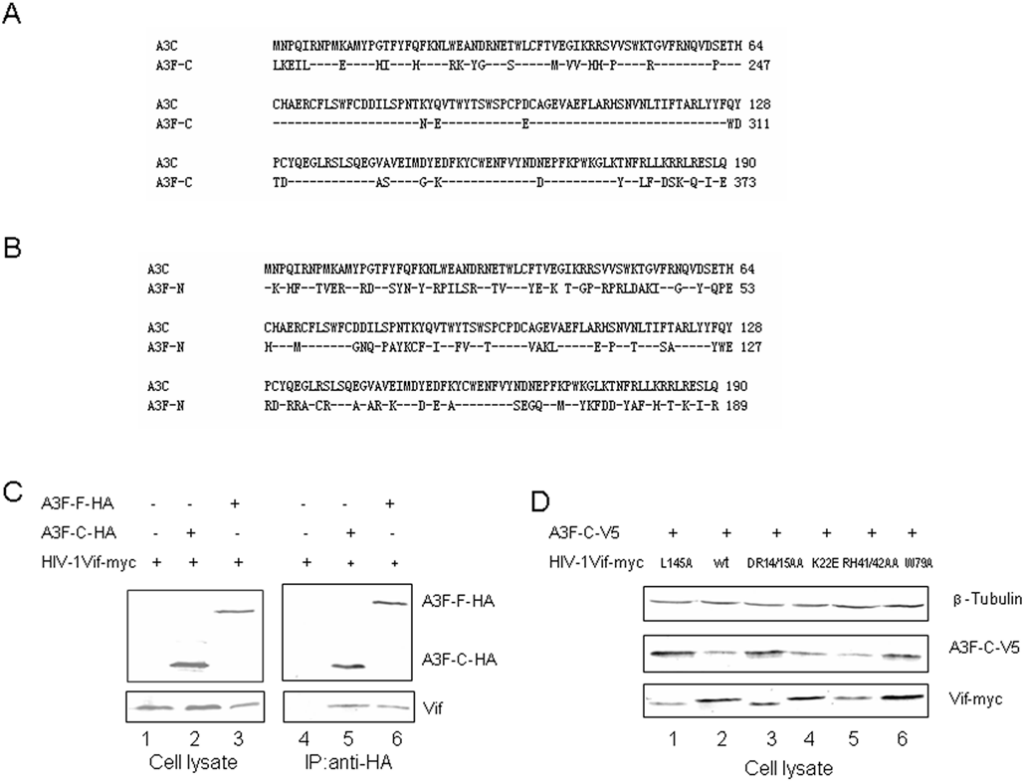
The C-CCD of A3F behaves like the full-length A3F. (A) Alignment of A3C and the C-CCD of AF. (B) Alignment of A3C and the N-CCD of AF. (C) Interaction of A3F and A3F-C with HIV-1 Vif. HEK293T cells were cotransfected with HIV-1 Vif-myc plus control vector, full-length A3F-HA, or A3F-C-HA. The cells were treated with 10 µM MG132 12 h prior to harvesting., and A3F-HA proteins were immunoprecipitated from cell lysates with an anti-HA antibody conjugated to agarose beads. The interaction of A3F with HIV-1 Vif molecules was detected by immunoblotting with antibodies against A3F-HA and Vif antibody. (D) HIV-1 Vif induces the degradation of the C-CCD of A3F. HEK293T cells were transfected with an expression vector encoding the C-CCD of A3F plus a control vector, WT Vif, or one of the indicated Vif mutant expression vectors. A3F-C stability was assessed by immunoblotting with antibodies against V5, Vif-myc, and β-tubulin as a loading control.

HIV-1 Vif utilizes similar regions to interact with A3C, A3DE, and A3F. It is interesting to note that HIV-1 Vif suppresses the single-domain cytidine deaminase A3C and the double-domain enzyme A3F through similar means. This result raises the question of whether HIV-1 Vif recognizes the amino- or carboxyl- terminal domain of A3F. Alignment analysis of A3C and A3F showed that there is a strikingly higher degree of homology (77%) between A3C and the carboxyl-terminal domain of A3F (Fig. 6A) than with the amino-terminal domain of A3F (47%, Fig. 6B).

To determine whether the carboxyl-terminal domain of A3F alone can interact with HIV-1 Vif, we constructed an expression vector for the HA epitope-tagged carboxyl-terminal domain of A3F (amino acids 190–373). HEK293T cells were co-transfected with expression vectors for HIV-1 Vif-myc plus a control vector or either full-length A3F-HA or the carboxyl terminal A3F-C-HA. The A3F-HA and A3F-C-HA proteins were immunoprecipitated from transfected cell lysates with anti-HA antibody conjugated to agarose beads. Both full-length A3F-HA and A3F-C-HA (Fig. 6C, lanes 5 and 6) efficiently co-immunoprecipitated Vif-myc. Vif-myc was not co-immunoprecipitated in the absence of any A3F proteins (Fig. 6B, lane 4), indicating the specificity of the assay system.

Since the carboxyl-terminal domain of A3F alone could interact with HIV-1 Vif, we asked whether the A3F-C alone could be degraded by HIV-1 Vif. HEK293T cells were transfected with an expression vector for A3F-C-V5 plus a control vector (Fig. 6D, lane 1), the expression vector for WT Vif-myc (lane 2), or one of various Vif mutants, as indicated (lane 3, 4, 5, and 6). The intracellular level of A3F-C-V5 was efficiently reduced by WT Vif (Fig. 6D, lane 2) when compared to the vector control (Fig. 6D, lane 1). However, VifDR14/15AA and VifW79A showed an impaired ability to reduce A3F-C-V5 expression (Fig. 6D, lanes 3 and 6) when compared to WT Vif. VifK22E and VifRH41/42AA retained their ability to degrade A3F-C-V5 (Fig. 6D, lanes 4 and 5). These data indicate that the requirement for the degradation of full-length A3F is the same as for the degradation of the carboxyl-terminal domain of A3F.

## Discussion

Human APOBEC3 cytidine deaminases are either single-domain or double-domain proteins. Regions in the double-domain A3G protein that are important for HIV-1 Vif-mediated degradation have been shown to span both the amino- and the carboxyl-terminal domains of A3G. Conticello *et al*. has demonstrated that amino acids 54–124 of A3G alone can interact with HIV-1 Vif [68]. Residues D128 and D130 were later reported to be important for HIV-1 Vif binding as well [87]–[91]. More recently, Zhang *et al.* have demonstrated that although amino acids 1–156 of A3G are sufficient for the interaction with HIV-1 Vif, additional regions spanning amino acids 105–245 are required for HIV-1 Vif-mediated polyubiquitination and degradation [85]. Thus, the amino-terminal domain of A3G mediates its interaction with HIV-1 Vif, but both cytidine deamination domains of A3G are involved in Vif-mediated degradation.

We have now found that, unlike the case for A3G, the carboxyl-terminal domain alone of another double-domain protein, A3F, is sufficient for the interaction with HIV-1 Vif, and, more importantly, is all that is required for A3F to undergo Vif-mediated degradation. These new data regarding the degradation of A3F are consistent with our previous observations that the carboxyl- but not the amino-terminal domain of A3F is important for its functional interaction with HIV-1 Vif [71], [79]. Mutation of the aspartate residue, D128, in the amino-terminal domain of A3G has been shown to influence its recognition by HIV-1 Vif [87]–[89]. The analogous residue in A3F, E127, is not important for HIV-1 Vif binding [71], and modifications of this amino acid in human A3F do not change its recognition by HIV-1 Vif or SIVagm Vif [71]. Furthermore, while C-terminal tag modifications of A3F (HA-tag vs V5-tag) can significantly influence its ability to be degraded by HIV-1 Vif, the same modifications of A3G do not affect its ability to be recognized by HIV-1 Vif [79].

Here we also provide evidence that Vif-mediated degradation of the carboxyl-terminal domain of A3F is similar to that of full-length A3F. We and others have identified unique regions in HIV-1 Vif that are critical for A3G, but not A3F, degradation and vice versa [13], [67], [78]–[83]. For example, W11, D14, R15, and W79 of HIV-1 Vif are required for A3F, but not A3G, binding and degradation. On the other hand, K22, R41, and H42 of HIV-1 Vif are required for A3G but not A3F binding and degradation. Residues such as D14R15 and W79 in HIV-1 Vif that are important for full-length A3F binding and degradation are also important for Vif-mediated degradation of the carboxyl-terminal domain of A3F. On the other hand, residues such as K22 and R41H42 that are important for the Vif-mediated degradation of A3G are dispensable for Vif-mediated degradation of both full-length A3F and the carboxyl-terminal domain of A3F. These data indicate the carboxyl-terminal domain of A3F is the main target of HIV-1 Vif against A3F.

Our data also demonstrate that the N-terminal region of HIV-1 Vif mediates its binding not only to the target molecules A3G and A3F but also to A3C and A3DE. Interestingly, we found that the single-domain cytidine deaminase A3C is also recognized and degraded by HIV-1 Vif through a mechanism similar to that for A3F. The residues in HIV-1 Vif that are important for A3F interaction and degradation were also important for Vif-mediated degradation of A3C. In contrast, those that are mainly important for the Vif-mediated degradation of A3G were found to be dispensable for Vif-mediated degradation of A3C. A3C has a high degree of amino acid homology to the carboxyl-terminal domain of A3F. However, unlike A3F, A3C has only weak anti-HIV-1 function [16], [18]. It is not clear whether HIV-1 Vif has evolved to disable A3F, and its ability to suppress A3C is merely an incidental consequence of the high degree of homology between A3C and the carboxyl-terminal domain of A3F. Whether A3C has anti-HIV-1 activity in certain HIV-1 natural target cells in vivo is an open question.

Consistent with a previous report [72], we found that A3DE has substantial anti-HIV-1 activity (Fig. 4). This protein is expressed in peripheral blood mononuclear cells [72] and macrophages (data not shown). Sequence alignment of A3DE and A3F shows a high degree of homology between these two molecules, and HIV-1 Vif residues required for A3F binding and suppression are also essential for A3DE binding and suppression (Figs. 4 and 5).

**Figure 7:**
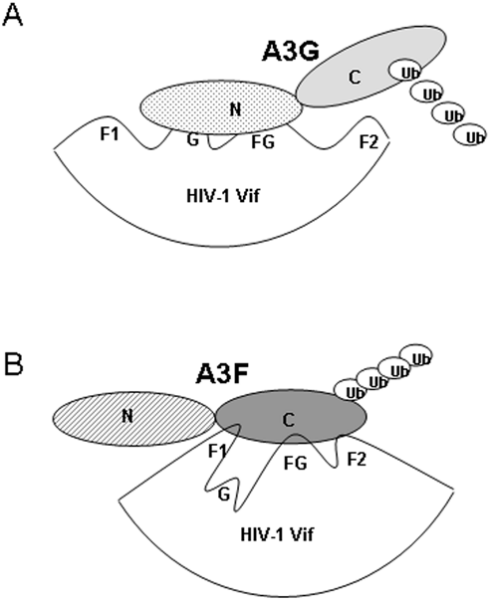
Models of HIV-1 Vif mediated interaction and polyubiquitination of A3G (A) and A3F (B). (A) Two distinct domains of HIV-1 Vif (G-box and FG-box) mediate interaction with the amino-terminal domain of A3G. However, the carboxyl-terminal domain of A3G is also required for Vif-mediated polyubiquitination and degradation. (B) Three distinct domains of HIV-1 Vif (F1-box, F2-box, and FG-box) mediate interaction with the carboxyl-terminal domain of A3F. The carboxyl-terminal domain of A3F is sufficient for Vif-mediated A3F degradation.

Collectively, our data suggest that Vif employs distinct protein interfaces to recognize various human APOBEC3 proteins (Fig. 7). HIV-1 Vif recognizes A3F, A3C, and A3DE through a similar mechanism that is distinct from that for A3G (Fig. 7). The W^11^xxDRMR^17^ and T^74^GERxW^79^ motifs of HIV-1 Vif are dispensable for the suppression of the potent anti-HIV-1 cytidine deaminase A3G yet are highly conserved among diverse HIV-1 strains. It is an open question whether these motifs have been conserved only for the suppression of A3F or whether they have been evolutionarily selected to target other human cytidine deaminases, such A3C and A3DE, as well. The highly conserved nature of these residues in diverse HIV-1 Vif molecules indicates that A3F, A3C, and/or A3DE represent a selection force against HIV-1 *in vivo*. This argument would be consistent with the *in vivo* observation of HIV-1 G-to-A mutation patterns. GA-to-AA mutations (a pattern generated by A3F, A3C, and/or A3DE) are frequently detected in viral sequences recovered from HIV-1-infected individuals [20]. The highly conserved residues in Vif that are required for the suppression of multiple human cellular anti-HIV-1 factors represent another potential drug target against HIV-1.

## Materials and Methods

### Plasmid construction

The infectious molecular clone of the Vif mutant pNL4-3ΔVif construct was obtained from the AIDS Research Reagents Program, Division of AIDS, National Institute of Allergy and Infectious Diseases (NIAID), National Institutes of Health (NIH). The SIVagmTanΔVif construct was prepared by inserting four nucleotides into the *Age* site in the *Vif* coding region of pSIVagmTan-1. VR1012, pHIV-1Vif-myc, pA3G-HA, and pA3F-HA have been previously described [62]. The A3C HA-tagged expression plasmid was a kind gift from Dr Michael Malim (Guy's, King's, and St. Thomas' School of Medicine, King's College, London). A3DE was a generous gift of Dr Y.H. Zheng (Biomedical and Physical Sciences, Michigan State University). Plasmids pVifDR14/15AA, pVifK22E, pVifRH41/42AA, and pVifW79A, were made from pHIV-1Vif-myc by site-directed mutagenesis. A3F-C-HA was amplified with the following primers: forward 5′- GTACGTCGACGCC ATG AACCCGATGGAGGCAATGTATC-3′, reverse 5′- GTACGCGGCCGCTCACGCGTAATCTGGGACGTCGTAAGGGTACTCGAGAATCTCCTGCAGCTTG -3′ containing *Sal*I and *Not*I sites, respectively, and a C-terminal HA tag. The PCR product was cloned into VR1012 to generate A3F-C-HA. A3F-C-V5 was constructed with the above forward primer and the reverse primer 5′- GTACGCGGCCGCTCACGTAGAATCGAGACCGAGGAGAGGGTTAGGGATAGGCTTACC CTCGAGAATCTCCTGCAGCTTG-3′ containing *Sal*I and *Not*I sites, respectively, and a C-terminal V5 tag. The PCR product was cloned into VR1012 to generate A3F-C-V5.

### Cell culture, transfection, viral infectivity (MAGI) assays, APOBEC3 degradation, and antibodies

HEK293T and MAGI-CCR5 (AIDS Research Reagents Program) cells were maintained and transfected or infected as previously described [62]. Transfection was performed with Lipofectamine2000 (Invitrogen) as instructed by the manufacturer. Viral infectivity (MAGI assays) were performed as described [62]:Virus was produced by transfecting HEK293T cells in a six-well plate with 1 µg of NL4-3△Vif or SIVagmTanΔVif, 1 µg of wild-type or mutant Vif, and 0.3 µg of APOBEC3 as indicated. Virus was harvested from the supernatant for viral infectivity assays, and cell lysates were prepared for immunoblotting. Infectivity was assessed at 48 h post-infection and normalized to the input CAp24 or CAp27. The antibodies used in this study have been previously described [84]: anti-HA antibody, anti-myc antibody, anti-Vif (AIDS Research Reagents Program), and anti-human ribosomal P antigens. The anti-Vif antibody was obtained from the AIDS Research Reagents Program (2221), the mouse anti-V5 antibody from Invitrogen (R96025), and the mouse anti-tubulin antibody from Covance (MMS-410P).

### Immunoprecipitation and immunoblot analysis

A T-25 flask of HEK293T cells was transfected with 3 µg APOBEC3 plus 3 µg of wild-type or mutant Vif expression vectors as indicated. Cells were harvested, washed twice with cold PBS and lysed in lysis buffer (50 mM Tris–HCl [pH 7.5] with 150 mM NaCl, 1% [v/v] Triton X-100, and complete protease inhibitor cocktail tablets) at 4°C for 1 h, then centrifuged at 10,000*g* for 30 min. For myc-tag immunoprecipitation, pre-cleared cell lysates were mixed with anti-myc antibody (Upstate) and incubated with protein G beads at 4°C for 3 h. For HA tag immunoprecipitation, pre-cleared cell lysates were mixed with anti-HA antibody-conjugated agarose beads (Roche) and incubated at 4°C for 3 h. Samples were then washed three times with washing buffer (20 mM Tris–HCl [pH 7.5], with 100 mM NaCl, 0.1 mM EDTA, and 0.05% [v/v] Tween-20). The beads were eluted with elution buffer (0.1 M glycine–HCl, pH 2.0), and 2× loading buffer was added. The eluted materials were then analyzed by SDS-PAGE and immunoblotting with the anti-myc antibody or the anti-HA antibody as described.
